# Functional outcome and complications after the microsurgical removal of giant vestibular schwannomas via the retrosigmoid approach: a retrospective review of 16-year experience in a single hospital

**DOI:** 10.1186/s12883-017-0805-6

**Published:** 2017-01-31

**Authors:** Xiang Huang, Jian Xu, Ming Xu, Mingyu Chen, Kaiyuan Ji, Junwei Ren, Ping Zhong

**Affiliations:** Department of Neurosurgery, Huashan Hospital, Fudan University, Shanghai, 200040 People’s Republic of China

**Keywords:** Vestibular schwannoma, Neurosurgical procedures, Postoperative complications, Facial nerve injuries

## Abstract

**Background:**

Intracranial vestibular schwannoma still remain to be difficulty for its unique microsurgical technique and preservation of neuro-function, as well as reducing common complications that may arise in surgery.

**Methods:**

We consecutively enrolled 657 unilateral giant (>4 cm diameter) vestibular schwannoma patients treated in Huashan Hospital via the suboccipital retrosigmoid approach in the past 16 years. The extension of tumor removal, surgical mortality, facial nerve function, hearing, and the other main short and long-term complications were the studied parameters.

**Results:**

Gross total resection was performed in 556 patients (84.6%); near-total resection was achieved in 99 patients (15.1%). The mortality rate is 0.6%. The main short-term complications included ‘new’ deafness (47.6%), intracranial infection (7.6%), lower cranial nerve defects (7.5%) and pneumonia (6.2%). The facial nerve was preserved anatomically in 589 cases (89.7%). Good facial nerve functional outcome (House-Brackmann Grades I and II) postoperatively was achieved in 216 patients (32.9%). Other 308 cases (46.9%) were House-Brackmann grade III, and 133 patients (20.2%) were House-Brackmann grade IV–VI. Follow-up data were available for 566 of the 657 patients (86.1%). The common long-term complications were hearing loss (85.2%), facial paralysis (HB grade IV–VI, 24.4%) and facial numbness (15.7%).

**Conclusions:**

Trends in the data lead the authors to suggest that the microsurgical technique, intraoperative nerve monitoring, and multidisciplinary cooperation, were the keys to improving prognostic outcomes in giant intracranial vestibular schwannoma patients.

## Background

The giant vestibular schwannoma still remains to be the difficulty for the surgical treatment of intracranial vestibular schwannoma now. The giant tumor would oppress and shift most cranial nerves in posterior cranial fossa, including oculomotor nerve, trochlear nerve, trigeminal nerve, abducens and lower cranial nerves, causing severe clinical symptoms [1]. It is difficult to identify the meager facial nerve and cochlear nerve clearly and preserve their function during operation because of giant tumor compression and its abundant blood supply. Moreover, surgical operation would affect the function of cerebellum and brainstem during treatment because of giant tumor, which lead to many complications after surgery. Thus, it is still a great challenge for the neurosurgeons to remove tumors totally, preserve neural function, reduce the complications of patients and improve long-term survival quality of life. Compared to others approaches, it is more beneficial to choose suboccipital retrosigmoid approach for the full exposure of vestibular schwannoma and preservation of facial and hearing functions [2]. Therefore, suboccipital retrosigmoid approach has been widely applied in our department. From 1999 to 2014, 657 cases of giant tumors, which were all resected trans suboccipital retrosigmoid approach, were obtained in our department. After the collection of clinical data, we conducted a retrospective review on clinical symptoms, surgical outcomes, distribution of complications, and their possible causes and preventive measures for the expectation of providing the reference for future clinical treatment.

## Methods

### Patient population

There were 657 consecutive giant unilateral VSs (excluding neurofibromatosis type II cases) surgically treated via suboccipital retrosigmoid approach between January 1999 and December 2014 at Our Hospital. Clinical status and complications were assessed postoperatively with 14 days and at the time of follow-up. The patients’ ages ranged from 12 to 80 years (mean age, 46.8 years). There were 289 male (44.0%) and 368 female (56.0%) patients. The peak incidence was between the ages of 40–60 years of age (366 cases, 55.7%) (Table 1). Patient charts were reviewed retrospectively for clinical status, operative findings, radiological results, and clinical outcomes. The data were analyzed with respect to tumor size, extent of resection, surgical results, and short- and long-term complications.

**Table 1:** Characteristics in 657 patients with intracranial giant vestibular schwannomas (*n* = 657)

Characteristics	
Age, year	
Range	12–80
Average	46.8
Sex, *N* (%)	
Female	289 (44.0%)
Male	368 (56.0%)
Extent of resection, *N* (%)	
Partial resection	2 (0.3%)
Near-total resection	99 (15.1%)
Gross total resection	556 (84.6%)
Managements during surgery	
Electrophysiological monitoring, *N* (%)	538 (81.9%)
External ventricular drainage, *N* (%)	15 (2.3%)
1/3 lateral cerebellum resection, *N* (%)	17 (2.6%)
Follow-up range, month	6–191
Average, month	59.6
Availability, *N* (%)	566 (86.1%)

### Tumor classification

Tumor size was categorized according to the international criteria using the largest extrameatal tumor diameter on the post-contrast axial magnetic resonance image (MRI). Giant tumors means >40 mm in the cerebellopontine angle (CPA) [3].

### Evaluation of facial nerve (FN)

FN function was evaluated according to the House-Brackmann (H-B) FN function grading scale immediately after surgery and at the time of last follow-up [4]. We classified FN function into 3 categories: good (H-B I + II), fair (H-B III), and poor (H-B IV + V + VI).

### Evaluation of hearing nerve

The hearing function was evaluated by audiology examinations including pure tone audiometry (PTA) and speech discrimination score (SDS). The criteria used for the preoperative and postoperative hearing evaluations were provided by the classification method of the American Institute of Otolaryngology-Head and Neck Surgery (AAO-HNS), 1995 [5].

### Surgical technique

The sub-occipital retrosigmoid approach was used in every case. Patients were settled in lateral position and a straight-incision behind ear was chosen. A surgical bone window was made to expose the connection of the transverse sinus and sigmoid sinus. The size of the craniotomy is independent of the tumor size because it is adjusted to the necessary cerebellar retraction. After dissecting the dural using a curved incision, the cerebrospinal fluid (CSF) was aspirated slowly from cerebellomedullary cistern to expose the tumor. Intraoperative electromyogram (EMG) monitoring was used in 538 cases (81.9%). EMG recordings of the orbicularis oris and oculi muscles were used to monitor FN function. A bipolar stimulus with an intensity of 1 mA to 0.05 mA and a duration of 0.1 ms was used to assess FN response.

### Multidisciplinary cooperation

**Fig. 1:**
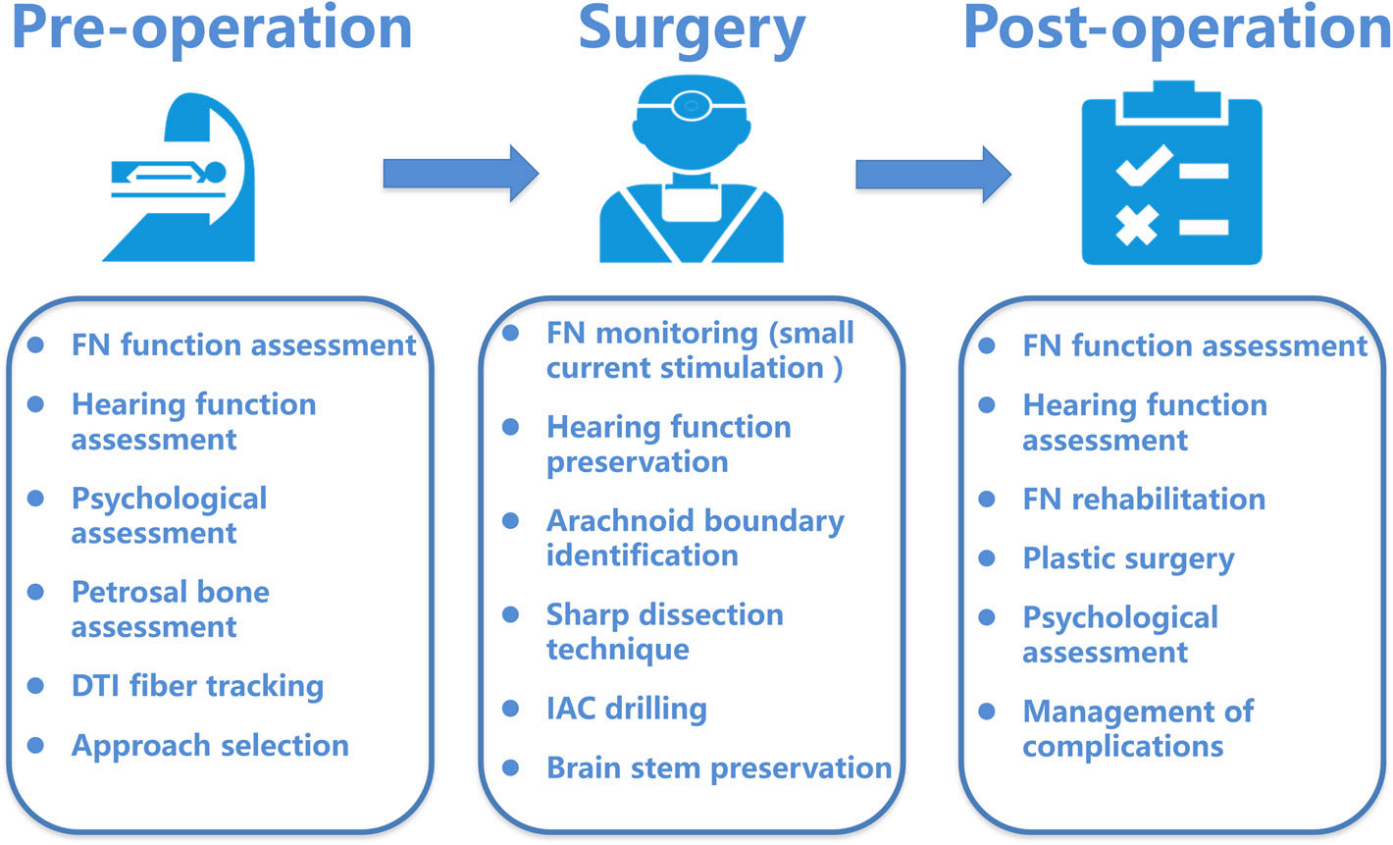
Multi-disciplinary cooperation treatment and surgical technique for giant vestibular schwannomas. FN: facial nerve, DTI: Diffusion tensor imaging, IAC: internal auditory canal

In recent 3 years, multidisciplinary cooperation strategy was used to treat giant vestibular schwannoma, which included diffusion tensor imaging (DTI) FN fiber tracking technology preoperatively, perioperative comprehensive assessment of facial and hearing function, perioperative psychological assessment, standard retrosigmoid approach technology, intraoperative EMG monitoring, facial function rehabilitation training and/or plastic surgery (Fig. 1).

### Follow-up investigation

All patients were evaluated with contrast-enhanced MRI follow-up from 3 months to 1 year after operation. If the tumor was totally removed or the residual tumor was proved to be stable, patients were re-investigated yearly for at least 5 years clinically and radiologically using MRI. For evaluation we checked for facial symmetry at rest and in motion, separate movements of all three segments (forehead, eyes and mouth), and incidence of conjunctivitis and synkinesis, then graded the results by the H-B scale. If the patient could not come to the hospital a questionnaire was mailed to him/her 1 year postoperatively, questioning his/her perception of the functional recovery, as compared with the clinician’s observation.

### Analysis of postoperative complications

Postoperative complications were evaluated immediately perioperatively (within 2 weeks to detect short-term complications) and at the time of the last follow-up (to detect long-term complications). The results of FN outcome, hearing outcome, disequilibrium and tinnitus were reported, which were the important result intrinsic to the nature of the disease. Patients who complained of imbalance or unsteadiness after surgery were determined to have disequilibrium. Tinnitus is regarded as subjective and is therefore reported by patients. Facial numbness, taste disturbance, and lower cranial nerve deficits were categorized as cranial nerve complications. Other non-neurological complications included acute hydrocephalus postoperatively, hematoma in the brain, CSF leak and meningitis. Keratitis, labial herpes, occipital scalp hydrops and pneumonia were categorized as systemic complications. Categorical data were compared by the *χ*2 test with continuity correction if appropriate. Continuous variables were expressed as the mean ± SD, and were compared using Student’s *t*-test. Two-tailed *P*-values <0.05 were considered to indicate a statistically significant difference. Statistical analysis was performed with the Stata software package (version 10.0).

## Results

### Clinical manifestation

All of those 657 patients were noticed some degree of hearing deficit and 248 patients (37.7%) experienced deafness. The other most frequent clinical symptoms were facial paresthesia (453 cases, 68.9%), tinnitus (296 cases, 45.1%), disequilibrium (293 cases, 44.6%), facial paralysis (HB III + IV 204 cases, 31.1%).

### Surgical results

Of 657 cases of giant tumor, the external ventricular drainage from the occipital area was used in 15 cases (2.3%) before tumor resection because of hydrocephalus and 1/3 lateral cerebellum resection was used in 17 cases (2.6%). EMG monitoring was used in 538 cases (81.9%) during surgery. Gross total resection (GTR) was achieved in 556 cases (84.6%), near-total resection (NTR) was in 99 cases (15.1%) and only 2 cases (0.3%) had partial resection (Table 1). The FN anatomical preservation rate was 89.6%.

### Mortality

Four patients died after the operation totally. The mortality rate is 0.6%. Two patients died because of the brain hemorrhage and acute hydrocephalus. One patient died of brain edema. One patient died of severe pneumonia due to low cranial nerve disturbance after the operation.

### Follow-up outcome

Of 657 cases, 566 patients (86.1%) were successful followed up. Ninety-one patients (13.9%) were lost followed up. The follow-up range was 6 to 191 months (average 59.6 months).

### Surgical complications

Of 657 cases within 2 weeks after surgery, disequilibrium was found in 186 cases (28.3%), new tinnitus in 61 cases (9.3%) and new hearing loss (AAO-HNS grade D) in 313 cases (47.6%). In follow-up data available for 566 patients (long-term results), disequilibrium was improved and only found in 38 cases (6.71%). Tinnitus was also improved and found in 14 cases (2.47%). However, loss of hearing (AAO-HNS grade D) seem to be not improved in long-term results and was found in 482 cases (85.2%). Other 67 patients (11.8%) were in grade C, 17 patients (3.00%) in grade B. Permanent facial paralysis (House-Brackmann III + IV + V) was in 250 cases (44.2%).

Other main short-term postoperative non-neurological complications included hematoma in 8 cases (1.2%), meningitis in 50 cases (7.6%), CSF leak in 18 cases (2.7%). The cranial nerve complication included lower cranial nerve deficit in 49 cases (7.5%). The systemic complications included keratitis in 8 cases (1.2%), labial herpes in 42 cases (6.4%), occipital scalp hydrops in 3 cases (0.5%) and pneumonia in 41 cases (6.2%).

However, for the main long-term complications, the condition was completely different. The incidence rate of long-term complications ranked from great to small as follows: facial numbness in 89 cases (15.7%), chronic headache in 18 cases (3.18%) and taste disturbance in 11 cases (1.94%), scar pain in 10 cases (1.77%), decreased vision in 7 cases (1.23%), delayed hydrocephalus in 6 cases (1.06%).

### FN function

#### Short-term FN outcome

Of 657 patients, the facial nerve was preserved anatomically in 589 cases (89.7%). Immediately after surgery (within 2 weeks), good (H-B grade I or II) FN function was seen in 216 cases (32.9%), fair (H-B grade III) FN function was seen in 308 patients (46.9%), and poor FN function (H-B grade IV-VI) was seen in 133 patients (20.2%).

In 556 cases of GTR group, good (H-B grade I or II) FN function was seen in 170 cases (30.6%), fair (H-B grade III) FN function was seen in 271 patients (48.7%), and poor FN function (H-B grade IV–VI) was seen in 115 patients (20.7%).

In 99 cases of NTR group, 45 cases (45.5%) had good (H-B grade I or II) FN function, 36 cases (36.4%) had fair (H-B grade III) FN outcome, 18 cases (18.2%) had poor (H-B grade IV–VI) FN function outcome after surgery. Two patients received partial resection because their tumors were too large and had fair FN outcome (H-B grade III).

#### Long-term FN outcome

Of 566 patients who were successfully followed up after surgery, 316 cases (55.8%) had good (H-B grade I or II) FN function, 112 cases (19.8%) had fair (H-B grade III) FN outcome, 138 cases (24.4%) had poor (H-B grade IV–VI) FN function outcome in follow-up period.

#### The effect of multidisciplinary cooperation strategy

From 2012 to 2014, there were 154 patients received multidisciplinary cooperation strategy to treat giant vestibular schwannoma. The anatomical FN preservation rate is 98.1%. Immediately after surgery (within 2 weeks), good (H-B grade I or II) FN function was seen in 84 cases (54.5%), fair (H-B grade III) FN function was seen in 37 patients 24.0%), and poor FN function (H-B grade IV–VI) was seen in 33 patients (21.4%) (Table 2).

**Table 2:** The effect comparison of tradition and multidisciplinary cooperation group (*n* = 657)

Group	NO.	GTR (%)	FN preservation	FN function 2 weeks after surgery (%)		
			(%)	H-B I or II	H-B II	H-B IV–VI
Tradition	503	402 (79.9)	438 (87.1)	132 (26.2)	271 (53.9)	100 (19.9)
MDC	154	154 (100.0)	151 (98.1)	84 (54.5)	37 (24.0)	33 (21.4)
Total	657	556 (84.6)	589 (89.6)	216 (32.9)	308 (46.9)	133 (20.2)

*Abbreviations*: *MDC* multidisciplinary cooperation group, *FN* facial nerve, *GTR* gross total resection

### Hearing function

Before the operation, 248 patients (37.7%) suffered from grade D hearing loss, 101 patients (15.4%) suffered from grade C hearing loss, and 308 patients (46.9%) suffered from grade C hearing loss, according to the classification of AAO-HNS. After the surgical treatment, 561 patients (85.4%) lost hearing completely (AAO-HNS grade D), among them, 313 of whom were “new hearing loss” and were classified into grade B or C before operation. The other 74 patients (11.2%) were in grade C, 22 patients (3.35%) in grade B. The hearing preservation rate in terms of the results of patients who had serviceable hearing pre-operatively was 7.14%.

## Discussion

According to the literature review, although giant vestibular schwannoma accounts for only 2% of vestibular schwannoma, [6] the percentage of giant vestibular schwannoma is much higher in large neurosurgery center, such as German Hanover neuroscience center (12.5%) [7]. From 1999 to 2014, 1263 cases of vestibular schwannomas were treated in our department and giant tumor accounts for 52%(657 cases). One reason was that patients were comparatively centralized because Huashan hospital was a large neurosurgery center in China and the other was that it is not easy to diagnose vestibular schwannoma at its onset especially in developing country. Large or giant vestibular schwannoma was dominant in regions lacking checking means. So, More attention should be paid to the treatment of giant vestibular schwannoma.

### The surgical strategy for giant vestibular schwannoma

We have reported that clinical manifestations of giant vestibular schwannoma were not only induced by the oppression of facial and auditory nerves from the tumor but also were caused by the oppressive effects of cranial nerves, cerebellum and brainstem from the tumor based on the conclusion of 1009 cases of large vestibular schwannoma [8]. Microsurgery was still an optimum choice for the treatment of giant vestibular schwannoma. The objective of the operation was not only to maximum extent more than tumor removal but also to protect the function of facial and auditory nerves and other cranial nerves and reduce complications. The mortality of operation obviously decreased with the rapid development of minimal invasive neurosurgery technology and electrophysiological monitor. For giant vestibular schwannoma, operative difficulty increased as the tumor size increased and there existed some problems including low rate of GTR and poor nervous function preservation [7, 9–12]. So, some researchers proposed different strategies such as multi-step operations and part removal of the tumor supplemented by stereotactic radiotherapy [13–15]. We thought both multi-step operations and different treatments in stages were supplementary approaches for unsatisfactory effects of single operation. If doctors could improve surgical outcome, single operation still was an optimum choice. In this study, 657 cases all adopted single operation of microsurgery. The anatomy preservation rate of facial nerves was 89.7%. The rate of GTR was 84.6%. The mortality was only 0.6%. In recent 3 years, we conducted multidisciplinary cooperation and improved microsurgical technology. We implemented the intraoperative monitoring of multiple groups of the cranial nerves and strengthened postoperative facial nerve function rehabilitation. There were 154 cases Involved, the rate of GTR: 100%. The mortality: 0%. The anatomy preservation rate of facial nerves increased to 98.1%. The facial nerve function preservation rate 2 weeks after operation: 78.5% (H-B I–III stage). All indicated that single operation could reach satisfactory effects.

#### The choice of surgical approach

So far, there were several surgical approaches for giant vestibular schwannoma including suboccipital retrosigmoid approach, middle cranial fossa approach and extended trans-labyrinthine approach. Middle cranial fossa approach seems more suitable for small and medium vestibular schwannoma. Extended trans-labyrinthine approach damaged the audition and its anatomy preservation rate of facial nerves was 74.6–87.8% [2, 16]. Suboccipital retrosigmoid approach was convenient and safe for neurosurgeons. It could provide spacious view of cerebellopontine angle and expose brainstem and cerebellopontine angle structure quickly, safely with multi-aspects. The operator could look directly at anterior inferior cerebellar artery (AICA) and facial and auditory nerves. Hence, it was an optimum approach for protecting intracranial segments of facial nerve. It has been reported that using suboccipital retrosigmoid approach, the anatomy preservation rate of FN was 86–92%. The function retention rate: 72–75% [1, 7]. In this study, 657 cases all adopted suboccipital retrosigmoid approach. The anatomy preservation rate of FN: 89.7%. The rate of GTR: 84.6%. In recent 3 years, the anatomy preservation rate of FN increased to 98.1%. The rate of GTR: 100%. The facial nerve function retention rate 2 weeks after operation: 78.5% (House-Brackmann I–III stage). We thought suboccipital retrosigmoid approach was appropriate for total removal of giant vestibular schwannoma.

#### Key operation points of suboccipital retrosigmoid approach

##### Exposure, decompression within the tumor and isolating tumor:

During operation, the cerebrospinal fluid should be released slowly after open the dura. After retracting the cerebellum the tumor was exposed and its arachnoid boundary should be conducted sharp dissection. Resected tumor piece-by-piece and conducted sufficient intra-tumor decompression. Meanwhile, closely guarded subarachnoid space. Then separated tumor from its upper to lower pole and then the section of brainstem carefully and separated and protected petrosal vein appropriately. Key operation points should be noted during operation because of giant tumor: ① The decompression speed cannot be too fast during operation (either when released cerebrospinal fluid of cisterna magna or when resected tumor) to avoid bleeding of bridging veins. ② Intraoperative hemostasis should be immediate and effective and keep clear operation field to separate the tumor arachnoid boundary. Paid more attention to the areas close to brainstem segments of auditory nerve and trigeminal nerve, contiguous areas of tumor and the choroid plexus of the fourth ventricle and the brainstem ventral veins. Arachnoid boundary was not clear and needed to repeat confirmation before separation. ③ Intra-tumor decompression should be sufficient during operation. Then removed the tumor wall gradually. After sufficient decompression, thinner tumor wall (2–3 mm) was easier to be separated from arachnoid interval, which was more beneficial to identify and protect facial and auditory nerves. ④ Close protected petrosal vein during operation because the injury of petrosal vein could lead to bleeding directly and the obstacles of cerebellum backflow, further contributing to tissue edema and/or the hemorrhage induced by venous congestion. ⑤ Conducted thorough hemostasis for the broken blood vessels during operation and avoided hemorrhage after operation. Paid more attention to AICA. Sughrue et al. summarized and analyzed 32,870 cases of vestibular schwannomas from 100 articles. The statistical results showed that the most common cause to death of vestibular schwannoma was AICA hemorrhage [11]. Separated and retained all blood vessels as much as possible when we removing the tumor. The arteries or veins on the surface of the tumor should be retained if they did not supply tumor, which could avoid affecting blood supply of peripheral nerve tissues and venous reflux.

##### The search and preservation of FN:

Auditory nerve would be firstly observed generally at the lower pole of tumor and should be assessed the reservation possibility. Tried to preserve the nerves if possible. The brainstem segment of FN could be found under the auditory nerve. Separated according to the distribution of nerves. Conducted sharp separation on the adhesion and tried to fully expose FN.

##### The management of internal auditory canal (IAC):

Drilled and opened the IAC and exposed the tumor inside. Resected the tumor while separated and preserved FN. Finally, closed IAC appropriately. It might be not necessary or unable to open IAC in the following situations: ① There was no tumor in IAC. ② The opening of IAC was enlarged by the tumor and it was not necessary to be drilled. ③ There were nerves and vessels on the surface of the tumor at the opening of IAC and it was difficult to separate. ④ There was high jugular bulb or Overdeveloped mastoid air cells.

##### The hemostasis and close:

Conducted precise hemostasis and close sutured dura and each layer of tissue.

### FN preservation

The favorable postoperative function of FN was achieved in 47.6 to 92.8% of vestibular schwannoma patients trans-suboccipital retrosigmoid approach, even lower in giant tumors [8, 17–24]. In this study, 589 cases (89.7%) showed preserved FN. There were 216 cases (36.2%) showed good FN outcome 2 weeks after surgery, 308 cases (45.8%) showed fair prognosis. In 566 cases who were available for follow-up, most patients showed recovered function of FN. The favorable function of FN (H-B I–III stage) was achieved in 86.0% of patients. The difficulty for FN preservation includes: the distinguishing of FN during operation; the preservation of FN during resection of tumor and the rehabilitation of FN function after surgery. Hence, we recommended that the preservation of FN should be improved by multidisciplinary cooperation. The specific highlights include: 1. The location relationship between tumor and FN should be assessed by imaging before surgery. 2. The microsurgical technology should be improved and monitoring of electrophysiology should be enhanced during surgery. 3. The plastic surgeon collaborates to do rehabilitation of FN function and repairing of FN.

### The location relationship between tumor and facial nerve should be assessed by imaging before surgery

DTI helps us to determine the diffusion tension of water molecules in white matter. DTI-based fiber tracking technology (DTT) helps us to visualize the direction where fiber bundle of the white matter in brain travels [25]. Many reports of vestibular schwannoma revealed that FN could be imaged and fiber tracking technology for FN has been considered as a novel, non-invasive accurate method to predict the location and direction of FN in large vestibular schwannoma. The success rate of DTT was reported to be 62.5–100% [26–28].

### The microsurgical technology should be improved and monitoring of electrophysiology should be enhanced during surgery

We believe that 2 key factors should be managed to preserve the FN during removal of tumor: 1. The blunt dissection should be combined with sharp dissection during dissection of FN. The FN themselves and blood supply should be preserved. 2. Accurate judgment of FN function during surgery: The EMG monitoring should be continuously given to determine the location and function of FN during surgery.

Some proposed to alternate the total resection with near-total or partial resection in order to preserve FN. However, Nonaka et al. [24] analyzed 410 cases of vestibular schwannoma and found that there was no significant difference in the anatomical and functional preservation of facial nerves in the GTR group and NTR group. In this study, 79.3% of 556 cases who underwent GTR showed good or fair prognosis of FN. 81.8% of 99 cases who underwent NTR showed good or fair prognosis of FN. Subsequent chi-squared analysis showed that good and fair FN outcomes did not correlate with the extent of resection (*P* = 0.569). We hence strengthened that preserving the FN at the condition of maximal resection of tumor. The continuous monitoring of electrophysiology should be performed to confirm the location of FN. Meanwhile, the decision on GTR or not should be made when the monitoring data of electrophysiology significantly change.

### Early rehabilitation of FN function and repairing of FN after surgery

Although we take many measures to preserve the FN, the preservation rate of FN is less than 100%. In this study, the anatomical preservation of FN was achieved in 589 cases (89.7%). What should be done to repair the ruptured FN? Sammi et al. [19, 29] suggested that the ruptured nerve should be immediately repaired if it was ruptured during the surgery. The methods to reconstruct the nerve included end-to-end anastomosis, intracranial-temporal bone nerve graft reconstruction and intracranial-extracranial graft reconstruction (intracranial-stylomastoid foramen). If the proximal and residual end of FN with impairment or complete degeneration is located in the brainstem, the hypoglossal nerve or contralateral FN can be used as donors to resuscitate the FN. The resuscitation should be done as early as possible. Early rehabilitation should be carried out after surgery.

Based on our experience, the ruptured nerve should be immediately repaired if it was ruptured during the surgery. The end-to-end anastomosis or nerve transplantation is the most common method. For patients with poor prognosis of FN function, if it was not possible to perform anatomical preservation during surgery, FN plastic surgery is recommend to repair the nerve (masseter nerve-facial nerve anastomosis). If the anatomical preservation was done during surgery, the strict rehabilitation should be strengthened and the recovery of FN function should be persistently observed. If the prognosis is poor, early intervention such as FN plastic surgery should be applied. For the time window of intervention, 1 year after surgery is the best period to predict whether the FN plastic surgery function is recovered or not (sensitivity 97%, specificity 97%) [30]. Therefore, we recommend early FN plastic surgery in the period of 1 year after tumor removal surgery for those who had poor prognosis despite anatomical preservation of facial nerves.

Among 566 cases who were successfully follow-up, 37 patients whose post-operative immediate FN function showed poor prognosis received only rehabilitation and recovered to HB I or II stage 1 year after surgery, 36 cases received masseter nerve-facial nerve anastomosis in plastic surgery and showed satisfactory outcome [31].

### Hearing preservation

The most relevant factor for preservation of hearing is the tumor size. The smaller tumor has the higher preservation possibility of hearing function after surgery. On the contrary, the larger tumor has the lower preservation possibility of hearing function after surgery [32]. Glasscock et al. [33] showed that patients with tumor bigger than 2 cm had highly low possibility of hearing preservation and early detection was critical for hearing preservation. We demonstrated that about 10% of patients with large vestibular schwannoma had hearing function [8]. For those patients, the acoustic nerve should be anatomically preserved with maximum effort. The preservation of hearing is still the goal of the surgery, which is dependent on normal internal auditory artery, structure of cochlea and cochlear nerve. Two problems should be solved to preserve the hearing: First, ensure the continuity of cochlear nerve and internal auditory artery. This requires the surgeon should have the ability to determine which one is cochlear nerve under the pathological state. Secondly, the effective monitoring of electrophysiology during the surgery is critical to complete preservation of cochlear nerve. The use of assistant-endoscopic surgery is helpful for preservation of hearing during resection of vestibular schwannoma. In this study, the hearing preservation rate was low because all the neuromas were giant. The preservation of hearing after surgery remains a highlighted issue for giant vestibular schwannomas.

### Other complications

Other complications included CSF leak, ataxia, tinnitus, facial numbness, taste disturbance, low cranial nerve deficit, post-operative hemorrhage, brain edema, meningitis etc. In this study, the incidence of CSF leakage is lower than literature report. Only 18 cases (2.7%) developed CSF leakage. However, CSF leakage is a common complication with incidence of 6.2–20% due to resection of large vestibular schwannomas, which is characterized by otorrhea, rhinorrhea and incision leakage of cerebrospinal fluid [34–39]. Sughrue et al. believed the most common factor for CSF leakage was associated with approach. It is not true that bigger tumors are more prone to leak of CSF. Instead, the trans-labyrinthine approach is more likely to cause CSF leakage than trans-retrosigmoid approach [11]. We concluded the following experience to avoid this complication: 1. It is best not to open the mastoid cells during operation. When it has to be open, bone wax and biogel should be used to rigorously seal it. 2. The opening of IAC shall not be excessively big. It should be big enough as long as the tumor in the canal is well exposed. During resection of tumor, the dura in the canal should be completely preserved. After resection of tumor, the canal should be carefully closed. 3. Strict suturing of dura is essential. The muscle and skin shall be rigorously sutured in organized layers. 4. The treatment of reducing intracranial pressure should be applied after surgery. The leakage of CSF should be immediately treated if it is happen. The mild incision leakage of CSF and scalp hydrops can be treated with subcutaneous puncture and pressure dressing. If it is not improved or it is suspected to be caused by poor closing of air chamber in the back wall of IAC, the exploration of CSF leakage and repairing procedures should be performed as soon as possible. For CSF rhinorrhea and otorrhea, cisternography or head MRI T2-weight imaging should be performed to confirm the location of leakage. The repairing should be immediately performed to avoid life-threatening complications such as intracranial infection caused by prolonged leakage of CSF. Meanwhile, the potent, sufficient antibiotics easy to pass blood brain barrier should be used to prevent and control infection. All cases with leakage of CSF in this study improved after treatment and discharged from the hospital.

In this study, 50 cases (7.6%) developed meningitis and the incidence was higher than literature report, which should be pay abundant attention to. Meningitis has two types, including bacterial meningitis and aseptic meningitis. The bacterial meningitis was more likely due to contamination during surgery, probably because of insufficient conformance with aseptic procedure and long surgery. The reasons for aseptic meningitis remained unknown. We found that the bacteria were often not considered in cases with meningitis after surgery of vestibular schwannomas. CSF samples were cultured for all the 50 cases of meningitis in this study. However, only 3 of them were bacteria-positive meningitis. Among these 3 cases, only 1 case was correlated with CSF leakage. Lebowitz et al. [40] suggested that most cases of meningitis after surgery of vestibular schwannomas were considered as aseptic meningitis and associated with filling the mastoid cells with bone wax. Therefore, we recommended the following key steps to reduce aseptic meningitis: 1. Carefully protect subarachnoid space, especially avoid hemorrhage during operation which flows into the surrounding subarachnoid space. 2. Reduce implants (such as glue, artificial dura matter, bone wax and hemostatic material), on the premise that the surgical outcome can be ensured. When closing cranium, each layer should be repeatedly washed with sterile normal saline to remove scraped bone substances and bone wax particles.

## Conclusions

To accumulate surgical experience and to master the clinical anatomy of the suboccipital retrosigmoid approach to vestibular schwannoma surgery, intraoperative nerve monitoring, preoperative study of the imaging and clinical data, and multidisciplinary cooperation, were the keys to improving prognostic outcomes in giant intracranial vestibular schwannoma patients.

## Abbreviation

AICA: Anterior inferior cerebellar artery
CPA: Cerebellopontine angle
CSF: Cerebrospinal fluid
DTI: Diffusion tensor imaging
DTT: Diffusion tensor imaging-based fiber tracking technology
EMG: Electromyography
FN: Facial nerve
GTR: Gloss total resection
H-B: House-Brackmann scale
IAC: Internal auditory canal
NTR: Near-total resection
STR: Subtotal resection
